# Whole-genome sequence-based comparison and profiling of virulence-associated genes of *Bacillus cereus* group isolates from diverse sources in Japan

**DOI:** 10.1186/s12866-019-1678-1

**Published:** 2019-12-16

**Authors:** Akiko Okutani, Satoshi Inoue, Akira Noguchi, Yoshihiro Kaku, Shigeru Morikawa

**Affiliations:** 1 Department of Veterinary Science, National Institute of Infectious Diseases, Tokyo, Japan; 2 Faculty of Veterinary Medicine, Okayama University of Science, Imabari, Ehime Japan

**Keywords:** *Bacillus cereus* group, Principal component analysis, Nonmetric multidimensional scaling, Whole-genome sequence, Epidemiological variables, Multilocus sequence typing, Core-genome SNP typing

## Abstract

**Background:**

The complete genome sequences of 44 *Bacillus cereus* group isolates collected from diverse sources in Japan were analyzed to determine their genetic backgrounds and diversity levels in Japan. Multilocus sequence typing (MLST) and core-genome single-nucleotide polymorphism (SNP) typing data from whole-genome sequences were analyzed to determine genetic diversity levels. Virulence-associated gene profiles were also used to evaluate the genetic backgrounds and relationships among the isolates.

**Results:**

The 44 *B. cereus* group isolates, including soil- and animal-derived isolates and isolates recovered from hospitalized patients and food poisoning cases, were genotyped by MLST and core-genome SNP typing. Genetic variation among the isolates was identified by the MLST and core-genome SNP phylogeny comparison against reference strains from countries outside of Japan. Exploratory principal component analysis and nonmetric multidimensional scaling (NMDS) analyses were used to assess the genetic similarities among the isolates using gene presence and absence information and isolate origins as the metadata. A significant correlation was seen between the principal components and the presence of genes encoding hemolysin BL and emetic genetic determinants in *B. cereus*, and the capsule proteins in *B. anthracis*. NMDS showed that the cluster of soil isolates overlapped with the cluster comprising animal-derived and clinical isolates.

**Conclusions:**

Molecular and epidemiological analyses of *B. cereus* group isolates in Japan suggest that the soil- and animal-derived bacteria from our study are not a significant risk to human health. However, because several of the clinical isolates share close genetic relationships with the environmental isolates, both molecular and epidemiological surveillance studies could be used effectively to estimate virulence in these important pathogens.

## Background

For the last 20 years, both human and animal cases of anthrax, the highly lethal disease caused by *Bacillus anthracis*, have thankfully been absent from Japan [1]. In addition, outbreaks of disease caused by pathogenic *Bacillus cereus* strains showing virulence characteristics similar to those of *B. anthracis* have been restricted to a limited number of locations around the world [2–4]. However, *B. anthracis* spores can lie dormant in soil for several decades [5], and anthrax cases were sporadically reported in cattle and pigs in Japan in the 1980s [1]. Therefore, because of the likely presence of residual spores in the environment, there is still a risk that anthrax may re-emerge in Japan. Soil is usually rich in bacteria belonging to the *B. cereus* group, of which *B. anthracis* is a member. As such, it is an important matter for public health to investigate whether spores belonging to *B. anthracis* or highly pathogenic *B. cereus* strains, which also pose a threat to human health, are present in Japanese soils.

The diversity of *B. cereus* is reflected by its virulence and life cycle in the environment [6]. Multilocus sequence typing (MLST) and virulence gene profiling analyses of the emetic toxin-producing *B. cereus* strains associated with food poisoning or nosocomial infection have been conducted in many countries [7]. In Japan, food poisoning caused by emetic toxin-producing *B. cereus* contamination of processed rice products has been reported [8] along with cases of diarrheal food poisoning [9]. There have also been several outbreaks of nosocomial *B. cereus* infection transmitted through contaminated hospital linen [10, 11]. Although *B. cereus* is characterized by high-level pathogenicity and lifestyle diversity, a detailed analysis of virulence-associated genes carried by *B. cereus* group bacteria isolated from soil, animal feces, and human patients with food poisoning or nosocomial infections across Japan has not been performed. A comprehensive study of the genetic diversity among environmental and clinical *B. cereus* group isolates in Japan is also warranted.

Therefore, to assess the prevalence of spores from the *B. cereus* group in soil, animal feces, and human patients with food poisoning or nosocomial infections and to measure the potential risk of the existence of pathogenic *B. cereus* group isolates to humans in Japan, we began collecting soil and animal feces samples and subsequently isolated *B. cereus* group strains from the collected samples. To date, neither *B. anthracis* nor *B. cereus* strains carrying *B. anthracis*-type virulence genes have been isolated. Our previous study suggested that many past anthrax cases could be attributed to animal products imported from overseas [1]. However, we have isolated members of the *B. cereus* group other than *B. anthracis* from the environmental samples, so it is important to examine whether any of these strains contain the virulence-associated genes that could make them a threat to human health. The virulence-associated genes tested in the present study included cytotoxin genes, hemolytic toxin genes and enterotoxin genes from the genomes of the *B. cereus* group, and capsule genes and toxin genes from the genome of *B. anthracis*. Carroll et al. [12] developed BTyper, a high-throughput computational tool for virulence-based classification of *B. cereus* group isolates using nucleotide sequencing data. BTyper was developed to identify anthrax-causing and emetic *B. cereus* group genomes and provide an output encompassing *rpoB* and *panC* typing results, average nucleotide identity, 16S rRNA classification, antimicrobial resistance, and virulence-associated gene profiles.

Thus, in the current study, we estimated the risk posed by the *B. cereus* bacterial group isolated in Japan to human health using molecular typing techniques and epidemiological information. We performed whole-genome sequencing on soil, animal, and clinical *B. cereus* group bacteria isolated in Japan to perform comparative genomic analysis using MLST and core-genome single-nucleotide polymorphism (SNP) data to investigate genetic diversity in these sample types. The relationships among the isolates were then determined using principal component analysis (PCA) with the R package and nonmetric multidimensional scaling (NMDS) based on the presence/absence of virulence-associated genes and epidemiological information for each strain using BTyper.

## Results

### Isolation of *B. cereus* group strains

Altogether, 35 soil sampling sites and four animal fecal samples from Japan were analyzed by us. The numbers of colonies on NGKG agar after the first incubation were 74.1 ± 13.8 colony forming units (CFUs)/g for soil and 28.3 ± 4.3 CFUs/g for animal feces. We performed single colony isolation and the 199 colonies from soil and 28 strains from animals we obtained from the first selection were biochemically tested. All the isolates were motile rods with hemolytic properties and all were positive for β-hemolysis; they also produced rough, white-gray colored colonies on sheep blood agar. They produced lecithinase and dehydrogenized peptone on NGKG agar. The isolates grew under both aerobic and anaerobic conditions after heating at 65 °C for 10 min. All the positive isolates were then tested for the presence of insecticidal parasporal inclusions by Ziehl-Neelsen staining. Altogether, eight isolates (cowbarnIn4, horsefeces35, cowsilo24, cowbarnIn5, cowbarnOut11, gfu2–1, cowbarnIn3, and cow1_2016) were seen to have produced crystal proteins by light microscopy. Therefore, the resulting parasporal insecticide-producing isolates were classified as *B. thuringiensis* and the inclusion-negative isolates were designated as *B. cereus*.

### Phylogenic analysis using MLST and core-genome SNP typing

**Fig. 1:**
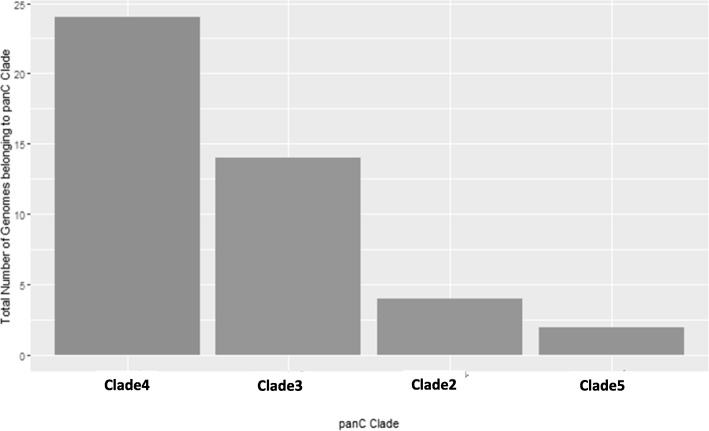
Closest-matching phylogenetic *panC* clade analysis of 44 Japanese *B. cereus* group isolates. The *panC* locus data from the 44 genome assemblies were analyzed using BTyper and BMiner [12]

**Fig. 2:**
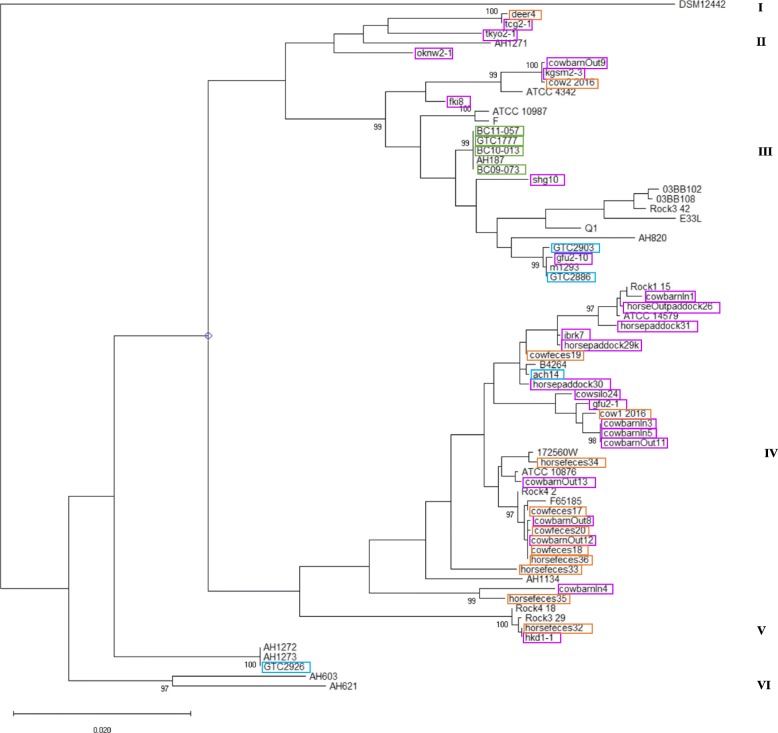
Maximum-likelihood phylogenetic tree generated using *B. cereus* group isolates and reference strains. The tree was constructed using MEGA X and the genetic distance from the Kimura 2-parameter model was inferred from the concatenated 2829-bp sequences of seven housekeeping genes examined during MLST of Japanese *B. cereus* isolates and *B. cereus* group isolates from countries other than Japan. The tree was drawn to scale, with branch lengths measured as the number of substitutions per site. All bootstrap support values > 95% (based on 1000 replicates) are shown next to the nodes. Clades I to VI are indicated. The sources of the Japanese isolates are indicated with colored rectangles: animal (orange), food poisoning (dark green), hospital (blue green), and soil (purple)

We carried out *rpoB* allelic and *panC* clade typing on 44 isolates using BTyper (Additional file 1: Table S1). Over half of the *B. cereus* group isolates we examined belonged to the *panC* clade IV (Fig. 1). We selected reference isolates from countries other than Japan including type strain ATCC 14579 (Additional file 3: Table S3) using criteria that covered all the *panC* clade from I to VII. Our concatenated MLST sequences were compared with those from 27 reference strains and a maximum-likelihood phylogenetic tree was constructed (Fig. 2). All the sequences clustered into six clades (I to VI) in branches with 1000 bootstrap values > 95% (Fig. 2). The Japanese isolates belonged to clades II–VI. Although clade IV contained the most Japanese isolates (Figs. 1 and 2), all the food poisoning isolates (BC09–073, BC10–013, BC11–057, GTC1777, and AH187) formed an independent branch for clade III (Fig. 2). The GTC2886 and GTC2903 inpatient isolates also clustered in clade III, whereas the ach14 and GTC2926 inpatient isolates belonged to clades IV and VI, respectively. Among the 25 isolates from the same farm environment in Ibaraki Prefecture, 22 (88%) clustered in clade IV.

**Fig. 3:**
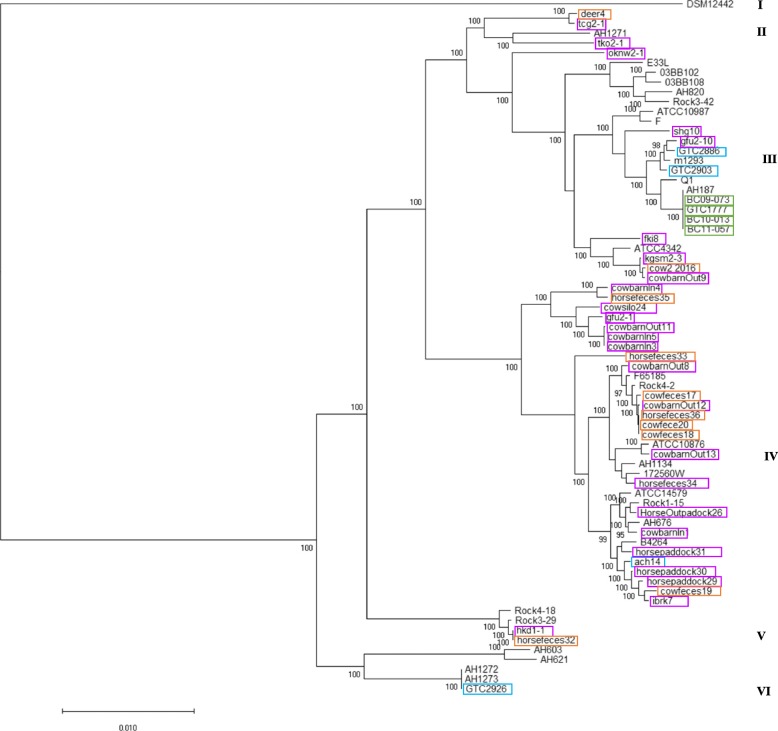
Phylogenic analysis using core-genome SNP typing. The phylogenetic tree is estimated from the core genome alignment generated by Parsnp using the Japanese isolates and *B. cereus* group isolates from countries other than Japan. The neighbor-joining algorithm was based on the core-genome SNP typing of the strains. Bootstrap confidence values were generated using 1000 permutations. The *B. cereus* ATCC 14579 strain (GenBank: NC_004722) was used as the reference genome in the analysis. The sources of the Japanese isolates are indicated by the following colored rectangles: animal (orange), food poisoning (dark green), hospital (blue green), and soil (purple)

The core-genome SNPs from the assembled genomes of the Japanese isolates and the strains from countries other than Japan were extracted and compared using the Parsnp tool [13] (Fig. 3). Strain cow1–2016 was omitted from the Parsnp alignment because its assembled genome was low quality with < 1% coverage of the reference genome and, despite it belonging to *panC* clade IV, its MLST sequence type (1573) and the ANIb result indicated that it belonged to *B. thuringiensis* (98.9%) (Additional file 2: Table S2). In common with the MLST phylogeny, all of the Japanese isolates belonged to *panC* clades II–VI (Figs. 2 and 3). Similar to the MLST results, 87.5% of the isolates (21 out of 24) from the same farm in Ibaraki Prefecture clustered in clade IV.

**Fig. 4:**
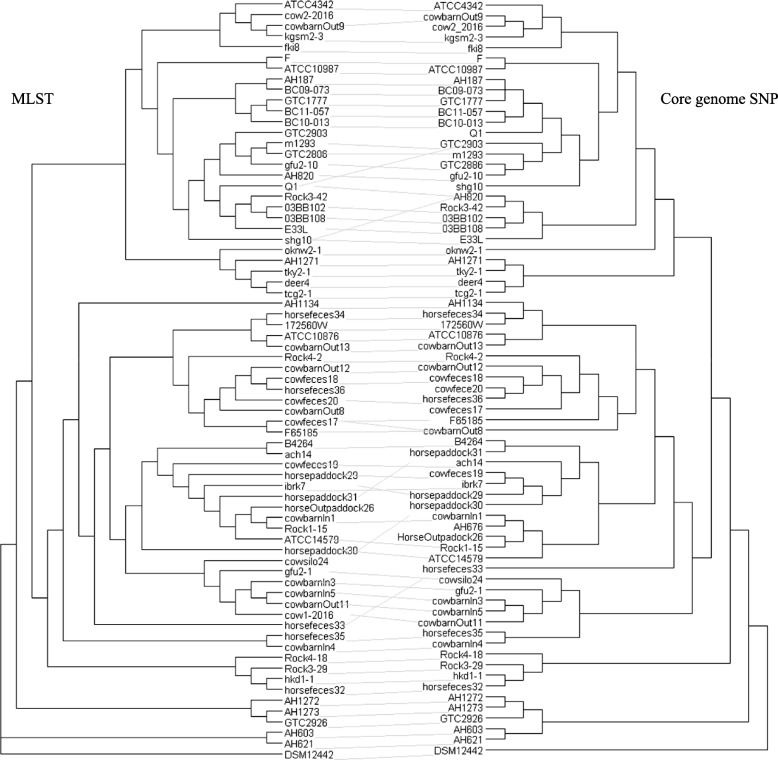
Tanglegram comparison between the core-genome SNP typing and the MLST phylogenies of *B. cereus* group bacteria. Tanglegram linking tips with the same label to each other via a straight line was produced within Dendroscope v3.6.3 for 71 *B. cereus* group isolates. The SNP-based neighbor-joining tree (right) was compared with the MLST-based maximum-likelihood tree (left). Clustering within the two trees was mostly congruent

Simpson’s diversity index analysis generated values of 0.9285 and 0.914 for the MLS types and core-genome SNP profiles among the strains, respectively. Our tanglegram analysis of the MLST and core-genome SNP phylogenic trees revealed that there was an almost congruent relationship between them (Fig. 4).

### PCA and NMDS analyses of Japanese *B. cereus* group isolates

We identified the virulence-associated gene profiles for the 44 isolates using BTyper (Additional file 1: Table S1). None of the isolates contained *B. anthracis*-associated *pag*, *cya*, and *lef* toxin genes, all of which are found on plasmid pXO1. The operon responsible for capsule formation in *B. anthracis*, *capBCDAE*, which is located on pXO2, was partially present in the genomes of the nosocomial infection-associated GTC2926 and GTC2886 strains, and in strain cowfeces17 also (Additional file 1: Table S1). Of the reads mapping to the capsule operon genes (Additional file 4: Table S4), we found that none of the isolates from which they originated possessed a complete operon for the capsule genes. As defined by ANIb (Additional file 2: Table S2) GTC2886 was defined as *B. paranthracis* (98.7%), GTC2926 as *B. paranthracis* (97.7%), and cowfeces17 as *B. cereus* (97.3%).

Genes, *cesA*, *cesB*, *cesC* and *cesD,* the genetic determinants of the encoding enzymes that assemble the emetic toxin, were only present in the genomes of isolates from the food poisoning cases, whereas *bpsE*, *cerA*, *nheA*, *sph*, *inhA2*, *clo*, *entA*, *plcR*, *nheC*, *nheB*, *cerB*, *inhA1*, *plcB*, and *bpsH* were present in the genomes of all the tested isolates. In contrast, *atxA* and *hasA* were absent from all the isolates.

**Fig. 5:**
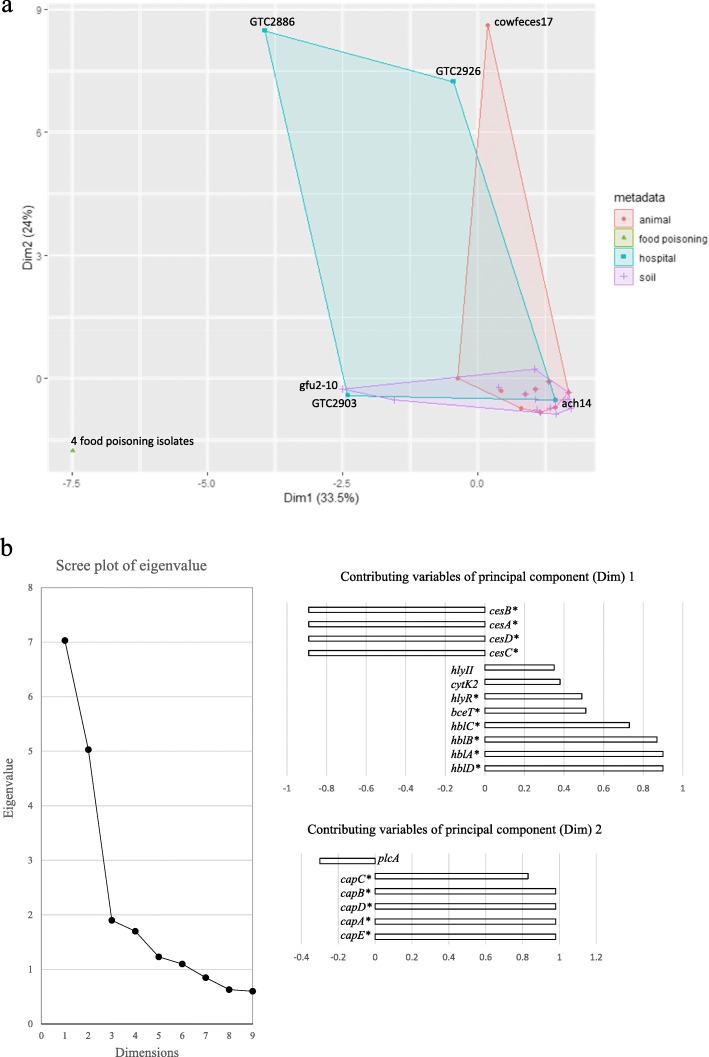
**a** Principal component analysis (PCA) of 44 Japanese *B. cereus* group isolates. The PCA was performed using the FactoMineR and Factoshiny packages (version 1.42) in R (version 3.5.2) software based on the presence/absence of virulence-associated genes determined by BTyper. Principal components 1 (Dim1) and 2 (Dim2) are plotted on the x and y axes, respectively. Each dot corresponds to an isolate. Isolates are clustered and colored by origin as animal (orange), food poisoning (dark green), hospital (blue green), and soil (purple), with all the isolates within a colored circle belonging to the same cluster. The variables factor map was generated according to cos2 > 0.7. **b** Scree plot of eigenvalue variance and the variables according to their contributions to principal components 1 (Dim1) and 2 (Dim2). Asterisks indicate *P* < 0.01. Variables significantly associated with a given principal component were calculated using the FactoMineR package (version 1.42)

To determine whether genotypic and phenotypic correlations exist among the isolates, PCA and NMDS analyses were performed. In the PCA, the correlation circles indicate strains that can be statistically grouped. Based on this analysis, the isolates from soil samples were grouped with the animal-derived ones (Fig. 5a). Interestingly, the scatter plot of the first two components (PC1 and PC2) showed that the hospital-associated cluster overlapped with both the animal- and soil-derived isolate clusters, and PC1 (Dim1: 33.47%) and PC2 (Dim2: 23.96%) both contributed to the PCA (Fig. 5a). Based on the eigenvalue variance values, the variables that significantly correlated with PC1 were the hemolysin BL (*hbl*) operon (*hblABCD*), *bceT*, *hlyR*, and the *ces* operon (*cesABCD*), whereas the *capBCADE* operon was significantly correlated with PC2 (Fig. 5b).

**Fig. 6:**
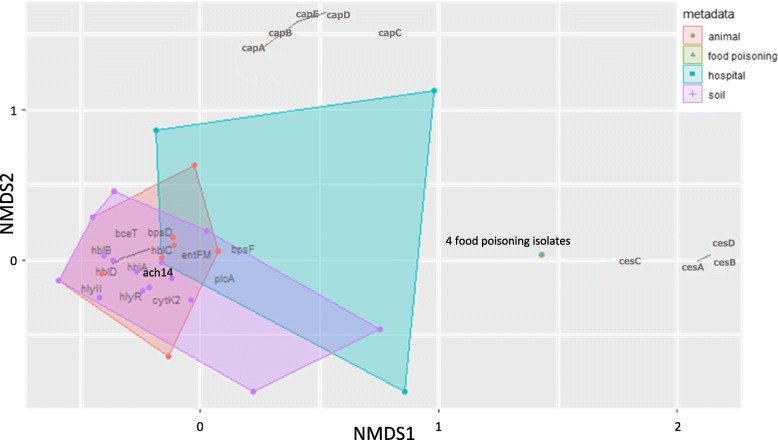
Nonmetric multidimensional scaling (NMDS) plot of 44 Japanese *B. cereus* group isolates. NMDS analysis was performed in BMiner using gene presence/absence data (Additional file 1: Table S1) and a Jaccard dissimilarity metric. Each dot corresponds to an isolate, and isolates are clustered and colored by origin as animal (orange), food poisoning (dark green), hospital (blue green), and soil (purple). All isolates located within a colored circle belong to the same cluster

The NMDS analysis showed that the animal cluster, which consisted mainly of isolates recovered from the same farm, and the soil cluster, which consisted of isolates collected from various locations around Japan, overlapped (Fig. 6). Three soil-derived isolates and five animal-derived isolates were included in the hospital-associated isolate cluster. Strain ach14 was located at the point where all the clusters overlapped, while all of the food poisoning isolates were plotted at the same point, separated from the other isolates.

## Discussion

In the present study, we analyzed the complete genome sequences of 44 *B. cereus* group isolates from diverse sources in Japan to investigate their genetic diversity and genetic backgrounds based on the presence or absence of virulence-associated genes. To compare these isolates, the whole-genome sequences from four *B. cereus* isolates from hospitalized human patients, four isolates recovered from the feces of patients with food poisoning, 10 isolates derived from soil, and 26 isolates derived from animals in Japan were used in a phylogenic analysis based on MLST and core-genome SNP typing. Whole-genome sequences from the *B. cereus* group isolates from non-Japanese countries were used for comparison with the Japanese isolates. Each of the genome sequences was analyzed to determine the *panC* clade grouping, the presence/absence of virulence-associated genes, and the *rpoB* type using BTyper [12]. We assigned the isolates to four different *panC* groups (Fig. 1) and five different clades by MLST and core-genome SNP analysis (Figs. 2 and 3). All of the food poisoning-derived isolates were assigned to sequence type ST26. This result is consistent with previous findings showing that emetic *B. cereus* group isolates belong to ST26 and *panC* clade III [14]. The tanglegram (Fig. 4) obtained using the MLST phylogeny with the concatenated sequences of seven genes (Fig. 2) or the core-genome SNP typing (Fig. 3) found no topological contradictions among the strains used in the present study. Together with the diversity values for the MLST and core-genome SNP typing calculated using Simpson’s index (0.9285 and 0.9142, respectively), the results suggest that the MLST analysis had sufficiently high power to discriminate the isolates and produced similar results to those of the core-genome SNP typing analysis (Fig. 4). Previous studies within other bacterial species, such as a study on the *Salmonella* serovar Enteritidis outbreak in Europe [15], and others on *Chlamydia trachomatis* [16], *Listeria monocytogenes* [17] and *Mycobacterium tuberculosis* [18], have shown congruent SNPs and MLSTs. In the present study, we were able to determine genetic diversity among Japanese isolates and isolates from countries other than Japan by phylogeny analysis using either MLST or core-genome SNP typing.

To identify any correlations among the isolates based on molecular and epidemiological information, PCA and NMDS were performed. The presence/absence of virulence-associated genes was converted to numeric variables and cluster information based on the sample origin represented the categorical variables. Most of the soil- and animal-derived isolates belonged to overlapping clusters (Fig. 5a and 6). Three of the four patient-derived isolates (GTC2886, GTC2903 and GTC2926) formed a cluster distinct from the soil and animal clusters, while hospital-derived strain ach14 overlapped with the soil and animal clusters. Soil isolates gfu 2–10 and GTC2903, which possess similar profiles in their virulence-associated genes, were neighbors in the plot (Fig. 5a). The food poisoning-derived isolates also clustered away from the other isolates. Genes in the *hbl* operon (*hlyR*, *bceT*) and genes in the *ces* operon were significantly correlated with PC1 (Fig. 5b). These findings are consistent with the results of Carroll et al. [12], who showed that the *hbl* operon was significantly associated with *B. cereus* phylogenetic clades. In addition, the *capBCDAE* operon was significantly correlated with PC2 in the current study (Fig. 5b). GTC2926, GTC2886, and cowfeces17, which were reported to possess genes within the *capBCDAE* operon by BTyper, were plotted with high scores on the PC2 axis. However, as Additional file 4: Table S4 shows, the read-mapping results for these genes revealed that only GTC2886 possesses all of the *capBCDAE* operon’s genes, but with low coverage percentages for *capA* (21.4%) and *capD* (7.9%). GTC2926 did not have any read coverage for the *capE* gene (Additional file 1: Table S1 and Additional file 4: Table S4), and cowfeces17 had no read coverage for the *capD* and *capE* genes (Additional file 4: Table S4), despite BTyper reporting the existence of these genes (Additional file 1: Table S1). While Kovac et al. [19] reported the presence of the *capBCDAE* operon in several *B. cereus* strains, along with a small number of *B. thuringiensis* and *B. toyonensis* strains, Carroll et al. [12] reported on *B. cereus* strains with partial possession of the *capBCDAE* operon. These results suggest that the *B. cereus* bacterial group of strains with anthrax-related virulence genes, such as capsule genes, should be analyzed to include coverage of its genes by gene mapping.

Our NMDS analysis revealed the same patterns as the PCA (Fig. 5a and 6), with the soil and animal clusters overlapping, and the hospital-derived strain ach14 overlapping with the soil, animal, and hospital clusters. This is interesting given that several cases of *B. cereus* infection caused by contaminated hospital linens or towels have been reported in Japan [10, 20]. We speculate that the *B. cereus* strains responsible for the contaminated hospital linen may have originated from an environmental source such as soil from outside of the hospital.

In the present study, we found a high level of similarity between *B. cereus* group bacteria isolated from environmental and clinical sources. We assessed the prevalence of *B. cereus* group bacteria in soil and animal feces, and in human patients with food poisoning or nosocomial infections also. *B. cereus* strains carrying *B. anthracis*-type capsule genes were found among the nosocomial strains and strains from the soil using BTyper. These results suggest that environmental *B. cereus* strains carrying *B. anthracis*-type capsule genes could be introduced into hospitals, with the potential risk of the transmission of pathogenic *B. cereus* group isolates to humans in Japan, so further investigation of this possibility is warranted. Some nosocomial infections are likely to occur following *B. cereus* contamination of the hospital environment as a result of human behavior. Therefore, to prevent opportunistic infections in a clinical setting, it is useful to adopt measures to minimize the transfer of bacteria from the external environment and to prevent their spread by thoroughly disinfecting any items likely to become contaminated (e.g. surfaces and linens) [21].

All of the isolates from the food poisoning cases contained the emetic cereulide toxin-encoding operon. Furthermore, because cereulide-positive *B. cereus* strains were isolated from 10.2% of the commercial foods distributed in Tokyo [22], further study is needed to investigate whether cereulide-positive strains isolated from commercial food products are genetically related to these food poisoning-associated isolates.

The number of isolates we used in the present study was too limited for a comprehensive analysis; however, our study’s data are consistent with the hypothesis that the possible source of nosocomial infections may be from contamination of the hospital environment, something that is potentially preventable by additional disinfection measures. Future work is needed to evaluate a larger number of isolates from each origin to confirm the observations made in the current study.

## Conclusions

The current MLST-based phylogeny and core-genome SNP typing analyses and the genetic features obtained by BTyper from our whole-genome sequences provide useful information for assessing the factors involved in the transmission of pathogenic *B. cereus* to humans from diverse resources. It has been suggested that strains isolated from soil and animals in Japan are unlikely to be a significant risk to human health. However, several of the clinical isolates from this study are closely related to environmental isolates, indicating that molecular surveillance with epidemiological features should be continued to aid in the implementation of effective measures against a possible resurgence in diseases caused by pathogenic *B. cereus* group bacteria, including anthrax or anthrax-like cases in Japan.

## Methods

### Isolation of the *B. cereus* bacterial group from soils and animals using selective media

All strains examined in the current study are listed in Additional file 2: Table S2. The number of sampling sites for soil was 35 and the number of animal fecal samples was 4. We performed single colony isolation and 199 strains from soil and 28 strains from animals from the first selection were subjected to the following biochemical tests to determine whether they were *B. cereus* group bacteria. Soil samples were collected between 2011 and 2018, with each sample weighing approximately 10 g and collected at a depth of 15–20 cm. Samples were collected from various locations in 20 different Japanese prefectures, including farms, beaches, parks, and bushland. Fecal samples were collected from deer in Yamaguchi Prefecture and from cows and horses on the Ibaraki University farm supported with Dr. Koji Uetsuka. All samplings were performed with permission of the owner or administrator of each location site.

To isolate the *B. cereus* bacterial group, approximately 250 mg of each sample was mixed with 0.5 ml of saline and vigorously vortexed before being heated at 65 °C for 10 min to kill all bacteria other than spores. Following centrifugation at 10,000×*g* for 5 min at room temperature, the supernatant was spotted onto the surface of selective NGKG (NaCl glycine Kim Goepfert) agar (Eiken Chemical Co., Tokyo, Japan) plates containing egg yolk, peptone, yeast extract, NaCl, glycine, 50,000 U of polymyxin B and phenol red, and then incubated at 30 °C for 20–24 h. Suspected *B. cereus* colonies, namely white colonies surrounded by dark pink agar (lecithinase positive and peptone dehydrogenization positive), were selected from each plate and sub-cultured on LB agar (BD Life Sciences, NJ, USA). The colonies were further identified by phenotypic and biochemical tests, including motility, hemolysis, culture under anaerobic conditions at 30 °C for 24 h with AneroPack (Mitsubishi Chemical, Tokyo, Japan), and cell shape with Gram staining under light microscopy. Glycerol stocks were prepared for all isolates and were maintained at − 80 °C. Candidate *B. cereus* group colonies from the LB agar plates were then cultured in LB broth and subjected to genomic DNA extraction using phenol [23]. To distinguish *B. thuringiensis* from *B. cereus*, parasporal insecticide production, which is unique to *B. thuringiensis*, was assessed by staining toxin crystals with Ziehl-Neelsen stain solution (Mutoh Kagaku, Tokyo, Japan). Briefly, the individual colonies cultured on LB agar plates were sub-cultured on nutrient agar and incubated overnight at 30 °C. The plates were then stored at room temperature for 2–3 days to lyse the vegetative cells, and the remaining colonies were spotted onto glass slides with a droplet of water. Following methanol fixation for 30 s, the spots were stained with Ziehl-Neelsen stain solution by warming for ~ 30 s. After rinsing under tap water, the free-form spores and toxin crystals were observed under light microscopy using a × 1000 oil-immersion lens (Nikon, Tokyo, Japan).

### Clinical *B. cereus* isolates from Japan

Three *B. cereus* isolates, BC09–073, BC10–013, and BC11–057, which were associated with food poisoning cases from Tokyo in 2009, 2010, and 2011, respectively, were kindly provided by Dr. Akiko Nakama of the Tokyo Metropolitan Institute of Public Health. GTC1777, an additional food-borne *B. cereus* isolate, and GTC2886, a nosocomial infection isolate were purchased from the NITE Biological Resource Center (NBRC, Tokyo, Japan). The whole-genome sequences from strain ach14 (which was isolated from a hospitalized patient in 2014 and kindly provided by Dr. Akihiro Nakao from Tsushima City Hospital in Aichi Prefecture) and *B. cereus* nosocomial infection strains GTC2903 and GTC2926 from NBRC have been reported previously [24].

### Whole-genome sequence analysis and phylogenetic tree construction using MLST and core-genome SNP typing

Genomic DNA libraries were prepared for each strain using the NEBNext DNA Library Prep Master Mix Set for Illumina (New England Biolabs; NEB, Ipswich, MA, USA) with NEBNext Multiplex Oligos for Illumina (Index Primers Set 1 and Set 2) (NEB) according to the manufacturer’s instructions. Libraries were then used for 2 × 151, 250, or 300 bp paired-end sequencing using the Illumina MiSeq platform (Illumina, San Diego, CA, USA) with a MiSeq Reagent Kit v2 (300 cycle, 500 cycle) or v3 (600 cycle).

The raw sequence data (fastq.gz files) were used for in silico MLST, from which the presence or absence of *B. anthracis* and *B. cereus* virulence-associated genes was determined, while *panC* clade typing using BTyper, a tool for virulence gene-based classification of *B. cereus* group isolates was also employed [12]. BTyper was designed for the virulence-based classification of toxin genes and capsule genes in *B. anthracis* and emetic toxin genes and other virulence-associated genes in *B. cereus.* The phylogenetic analyses conducted in MEGA X [25] were based on the concatenated sequences from the seven genes used for MLST [26]. The MLST analysis also used the concatenated sequences from 27 reference strains including Type strain ATCC14579 derived from countries other than Japan. These reference sequences were obtained from the MLST database for comparison with our isolates (https://pubmlst.org/bcereus/), and were selected to cover the *panC* clade from I to VII (Additional file 3: Table S3). Phylogenetic relationships were inferred using the maximum-likelihood method with MEGA X tools. Bootstrap scores were calculated from 1000 replicates for each phylogenetic reconstruction.

After filtering low-quality reads and quality trimming in CLC Genomics Workbench 11.0.1 (Qiagen) using the default parameters, de novo assembly of the high-quality paired-end reads was conducted using the CLC Genomics Workbench with standard settings. We used the Parsnp tool from the Harvest Suite software for fast multiple alignment of genomic sequences [26] using the *B. cereus* ATCC 14579 chromosome (NC_004722.1) as the reference genome. Assembled contigs were used as input for Parsnp v1.2 using the parameters -c and -C 1000. The detected SNPs were extracted to a VCF file, using HarvestTools v1.1.2 from the same software package and the phylogeny was visualized using gingr [13].

The total read count and average query coverage of the capsule operon (*capA*, *capB*, *capC*, *capD* and *capE*) in the three genomes from the *B. cereus* group isolates were calculated using the “mapped reads to reference” option in the CLC genomic workbench (version 11.0.1) with default parameters.

Simpson’s index of diversity was used to produce numerical values (D) to determine the discriminatory abilities of the typing schemes as per the equation described by Hunter and Gaston [27], where N represents the total number of strains (*N* = 71) and n_j_ represents the number of strains belonging to each typing sub-group.

Further comparison of the MLST and core-genome SNP typing was performed using the tanglegram algorithm [28], generated by Dendroscope v3.6.3 [29]. The tanglegram algorithm compares two phylogenetic networks by placing rooted trees side by side and drawing a straight line between corresponding taxa (identified through identical tip labels). The algorithm minimizes the number of crossings between connectors [28]; therefore, when two trees are identical no connectors will cross. Changes between the internal nodes of the phylogenies of the two trees can cause multiple short-range crosses, all in the same direction, between connectors; however, this would demonstrate that clustering at the tips of the phylogeny is the same.

### PCA and NMDS analyses based on molecular data and epidemiological information

Correlations among the isolates examined in the present study were visualized using PCA and NMDS [30]. PCA was used to identify correlations among the strains using the genetic information and data from the isolation sources generated in the current study and the FactoMineR and Factoshiny packages (version 1.42) in R (version 3.5.2) software (https://cran.r-project.org/). Profiles based on the presence/absence of virulence-associated genes were converted into quantitative variables, while strain origin information (soil, animal, hospital, or food poisoning) was used as the group options. NMDS was performed in BMiner using the gene presence/absence data and the Jaccard dissimilarity metric.

## Supplementary information

**Additional file 1: Table S1.** Presence or absence of *Bacillus cereus* virulence-associated genes in each genome sequence as detected using BTyper version 2.3.0 [12]. The profiles of virulence-associated genes from Japanese *B. cereus* group isolates are shown.

**Additional file 2: Table S2.** List of isolates, corresponding metadata, and accession numbers. The strain list for the Japanese *B. cereus* group isolates used in the present study is shown.

**Additional file 3: Table S3.** The 27 *Bacillus cereus* strains derived from countries other than Japan used in this study. The strain list for the *B. cereus* group isolates from countries other than Japan used in the present study is shown.

**Additional file 4: Table S4.** The capsule operon (*capA*, *capB*, *capC*, *capD* and *capE*) in three *B. cereus* group isolates genome. Total read count and average query coverage were calculated using “mapped reads to reference” option of CLC genomic workbench ver11.0.1 with default parameters. Total read count and query coverage of capsule operon are shown.

## Data Availability

The nucleotide sequence data for the isolates reported in this study have been deposited in the DDBJ Sequenced Read Archive under the accession numbers listed in Additional file 2: Table S2. All other data generated or analyzed during this study are included in this article.

## Abbreviations

MLST: Multilocus sequence typing
NMDS: Nonmetric multidimensional scaling
PCA: Principal component analysis
SNP: Single-nucleotide polymorphism

## References

[1] Okutani A, Inoue S, Morikawa S. Comparative genomics and phylogenetic analysis of *Bacillus anthracis* strains isolated from domestic animals in Japan. Infect Genet Evol. 2019;71:128–39. 10.1016/j.meegid.2019.03.022 30928604

[2] Antonation KS, Grutzmacher K, Dupke S, Mabon P, Zimmermann F, Lankester F, et al. *Bacillus cereus* biovar Anthracis causing anthrax in sub-Saharan Africa-chromosomal monophyly and broad geographic distribution. PLoS Negl Trop Dis. 2016;10:e0004923. 10.1371/journal.pntd.0004923 PMC5015827 27607836

[3] Klee SR, Brzuszkiewicz EB, Nattermann H, Brüggemann H, Dupke S, Wollherr A, et al. The genome of a *Bacillus* isolate causing anthrax in chimpanzees combines chromosomal properties of *B cereus* with *B anthracis* virulence plasmids. PLoS One. 2010;5:e10986. 10.1371/journal.pone.0010986 PMC2901330 20634886

[4] Hoffmaster AR, Hill KK, Gee JE, Marston CK, De BK, Popovic T, et al. Characterization of *Bacillus cereus* isolates associated with fatal pneumonias: strains are closely related to *Bacillus anthracis* and harbor *B. anthracis* virulence genes. J Clin Microbiol. 2006;44:3352–60. 10.1128/JCM.00561-06 PMC1594744 16954272

[5] Dragon DC, Rennie RP. The ecology of anthrax spores: tough but not invincible. Can Vet J. 1995;36:295–301. PMC1686874 7773917

[6] Ceuppens S, Boon N, Uyttendaele M. Diversity of *Bacillus cereus* group strains is reflected in their broad range of pathogenicity and diverse ecological lifestyles. FEMS Microbiol Ecol. 2013;84:433–50. 10.1111/1574-6941.12110 23488744

[7] Glasset B, Herbin S, Granier SA, Cavalie L, Lafeuille E, Guerin C, et al. *Bacillus cereus*, a serious cause of nosocomial infections: epidemiologic and genetic survey. PLoS One. 2018;13:e0194346. 10.1371/journal.pone.0194346 PMC5966241 29791442

[8] Agata N, Ohta M, Mori M, Isobe M. A novel dodecadepsipeptide, cereulide, is an emetic toxin of *Bacillus cereus*. FEMS Microbiol Lett. 1995;129:17–20. 10.1016/0378-1097(95)00119-P 7781985

[9] Kotiranta A, Lounatmaa K, Haapasalo M. Epidemiology and pathogenesis of *Bacillus cereus* infections. Microbes Infect. 2000;2:189–98. 10.1016/s1286-4579(00)00269-0 10742691

[10] Sasahara T, Hayashi S, Morisawa Y, Sakihama T, Yoshimura A, Hirai Y. *Bacillus cereus* bacteremia outbreak due to contaminated hospital linens. Eur J Clin Microbiol Infect Dis. 2011;30:219–26. 10.1007/s10096-010-1072-2 20938704

[11] Barrie D, Hoffman PN, Wilson JA, Kramer JM. Contamination of hospital linen by *Bacillus cereus*. Epidemiol Infect. 1994;113:297–306. 10.1017/s0950268800051724 PMC2271531 7925667

[12] Carroll LM, Kovac J, Miller RA, Wiedmann M. Rapid, high-throughput identification of anthrax-causing and emetic *Bacillus cereus* group genome assemblies using BTyper, a computational tool for virulence-based classification of *Bacillus cereus* group isolates using nucleotide sequencing data. Appl Environ Microbiol. 2017;83:e01096–17. 10.1128/AEM.01096-17 PMC5561296 28625989

[13] Treangen TJ, Ondov BD, Koren S, Phillippy AM. The harvest suite for rapid core-genome alignment and visualization of thousands of intraspecific microbial genomes. Genome Biol. 2014;15(11):524. 10.1186/s13059-014-0524-x PMC4262987 25410596

[14] Carroll LM, Wiedmann M, Mukherjee M, Nicholas DC, Mingle LA, Dumas NB, Cole JA, Kovac J. Characterization of emetic and diarrheal *Bacillus cereus* strains from a 2016 foodborne outbreak using whole-genome sequencing: addressing the microbiological, epidemiological, and Bioinformatic challenges. Front Microbiol. 2019;10:144. 10.3389/fmicb.2019.00144 PMC6379260 30809204

[15] Pearce ME, Alikhan NF, Dallman TJ, Zhou Z, Grant K, Maiden MCJ. Comparative analysis of core genome MLST and SNP typing within a European *Salmonella* serovar Enteritidis outbreak. Int J Food Microbiol. 2018;274:1–11. 10.1016/j.ijfoodmicro.2018.02.023. Epub 2018/03/27. PubMed PMID: 29574242; PubMed Central PMCID: PMCPMC5899760. 10.1016/j.ijfoodmicro.2018.02.023 PMC5899760 29574242

[16] Patino LH, Camargo M, Munoz M, Rios-Chaparro DI, Patarroyo MA, Ramirez JD. Unveiling the Multilocus Sequence Typing (MLST) Schemes and Core Genome Phylogenies for Genotyping *Chlamydia trachomatis*. Front Microbiol. 2018;9:1854. 10.3389/fmicb.2018.01854. Epub 2018/09/07. PubMed PMID: 30186244; PubMed Central PMCID: PMCPMC6113918. 10.3389/fmicb.2018.01854 PMC6113918 30186244

[17] Moura A, Criscuolo A, Pouseele H, Maury MM, Leclercq A, Tarr C, et al. Whole genome-based population biology and epidemiological surveillance of *Listeria monocytogenes*. Nat Microbiol. 2016;2:16185. 10.1038/nmicrobiol.2016.185. Epub 2016/10/11. PubMed PMID: 27723724. 10.1038/nmicrobiol.2016.185 PMC8903085 27723724

[18] Kohl TA, Diel R, Harmsen D, Rothganger J, Walter KM, Merker M, et al. Whole-genome-based *Mycobacterium tuberculosis* surveillance: a standardized, portable, and expandable approach. J Clin Microbiol. 2014;52(7):2479–86. 10.1128/JCM.00567-14. Epub 2014/05/03. PubMed PMID: 24789177; PubMed Central PMCID: PMCPMC4097744. 10.1128/JCM.00567-14 PMC4097744 24789177

[19] Kovac J, Miller RA, Carroll LM, Kent DJ, Jian J, Beno SM, Wiedmann M. Production of hemolysin BL by *Bacillus cereus* group isolates of dairy origin is associated with whole-genome phylogenetic clade. BMC Genomics. 2016;17:581. 10.1186/s12864-016-2883-z PMC4979109 27507015

[20] Dohmae S, Okubo T, Higuchi W, Takano T, Isobe H, Baranovich T, et al. *Bacillus cereus* nosocomial infection from reused towels in Japan. J Hosp Infect. 2008;69:361–7. 10.1016/j.jhin.2008.04.014 18602188

[21] Oie S, Furukawa H, Kobayashi H, Okubo T. Cleanliness of linen and clothing items professionally laundered or dry-cleaned. Jpn J Infect Dis. 2016;69:75–6. 10.7883/yoken.JJID.2015.007 26073734

[22] Arai T, Chiba T, Akiba T, Monma C, Nakama A, Kai A. Contamination of *Bacillus cereus* in commercial foods and the producibility of emetic toxin, cereulide, of the isolates (in Japanese). Ann Rep Tokyo Metr Inst Pub Health. 2012;63:173–9.

[23] Sambrook J, Russell DW. Purification of nucleic acids by extraction with phenol:chloroform. CSH Protoc. 2006. 10.1101/pdb.prot4455. 10.1101/pdb.prot4455 22485786

[24] Okutani A, Inoue S, Morikawa S. Draft genome sequences of three clinical strains of *Bacillus cereus* isolated from human patients in Japan. Microbiol Resour Announc. 2019. 10.1128/MRA.00415-19. 10.1128/MRA.00415-19 PMC6509534 31072885

[25] Kumar S, Stecher G, Li M, Knyaz C, Tamura K. MEGA X: Molecular evolutionary genetics analysis across computing platforms. Mol Biol Evol. 2018;35:1547–9. 10.1093/molbev/msy096 PMC5967553 29722887

[26] Priest FG, Barker M, Baillie LW, Holmes EC, Maiden MC. Population structure and evolution of the *Bacillus cereus* group. J Bacteriol. 2004;186:7959–70. 10.1128/JB.186.23.7959-7970.2004 PMC529064 15547268

[27] Hunter PR, Gaston MA. Numerical index of the discriminatory ability of typing systems: an application of Simpson’s index of diversity. J Clin Microbiol. 1988;26(11):2465–6. PubMed PMID: 3069867; PubMed Central PMCID: PMCPMC266921. 10.1128/jcm.26.11.2465-2466.1988 PMC266921 3069867

[28] Scornavacca C, Zickmann F, Huson DH. Tanglegrams for rooted phylogenetic trees and networks. Bioinformatics. 2011;27(13):i248–56. 10.1093/bioinformatics/btr210 PMC3117342 21685078

[29] Huson DH, Scornavacca C. Dendroscope 3: an interactive tool for rooted phylogenetic trees and networks. Syst Biol. 2012;61(6):1061–7. 10.1093/sysbio/sys062 22780991

[30] Ramette A. Multivariate analyses in microbial ecology. FEMS Microbiol Ecol. 2007;62:142–60. 10.1111/j.1574-6941.2007.00375.x PMC2121141 17892477

